# Drought Has a Greater Negative Effect on the Growth of the C_3_ *Chenopodium quinoa* Crop Halophyte than Elevated CO_2_ and/or High Temperature

**DOI:** 10.3390/plants13121666

**Published:** 2024-06-16

**Authors:** Zulfira Rakhmankulova, Elena Shuyskaya, Maria Prokofieva, Kristina Toderich, Luizat Saidova, Nina Lunkova, Pavel Voronin

**Affiliations:** 1 K.A. Timiryazev Institute of Plant Physiology of Russian Academy of Science, 127276 Moscow, Russia; zulfirar@mail.ru (Z.R.); evshuya@gmail.com (E.S.); maria.vdovitchenko@gmail.com (M.P.); saidovaluizat@mail.ru (L.S.); nina.lunkova@gmail.com (N.L.);; 2 Graduate School of Bioresources, Mie University, 1577 Kurimamachiya, Tsu 514-8507, Japan

**Keywords:** photosynthesis, individual factors, combined stress, protective mechanisms, PSI cyclic electron transport, drought, quinoa

## Abstract

Plant growth and productivity are predicted to be affected by rising CO_2_ concentrations, drought and temperature stress. The C_3_ crop model in a changing climate is *Chenopodium quinoa* Willd—a protein-rich pseudohalphyte (Amaranthaceae). Morphophysiological, biochemical and molecular genetic studies were performed on quinoa grown at ambient (400 ppm, aCO_2_) and elevated (800 ppm, eCO_2_) CO_2_ concentrations, drought (D) and/or high temperature (eT) treatments. Among the single factors, drought caused the greatest stress response, inducing disturbances in the light and dark photosynthesis reactions (PSII, apparent photosynthesis) and increasing oxidative stress (MDA). Futhermore, compensation mechanisms played an important protective role against eT or eCO_2_. The disruption of the PSII function was accompanied by the activation of the expression of *PGR5*, a gene of PSI cyclic electron transport (CET). Wherein under these conditions, the constant Rubisco content was maintained due to an increase in its biosynthesis, which was confirmed by the activation of *rbcL* gene expression. In addition, the combined stress treatments D+eT and eCO_2_+D+eT caused the greatest negative effect, as measured by increased oxidative stress, decreased water use efficiency, and the functioning of protective mechanisms, such as photorespiration and the activity of antioxidant enzymes. Furthermore, decreased PSII efficiency and increased non-photochemical quenching (NPQ) were not accompanied by the activation of protective mechanisms involving PSI CET. In summary, results show that the greatest stress experienced by *C. quinoa* plants was caused by drought and the combined stresses D+eT and eCO_2_+D+eT. Thus, drought consistently played a decisive role, leading to increased oxidative stress and a decrease in defense mechanism effectiveness.

## 1. Introduction

Extreme climate events are becoming more common and severe as global warming progresses. Heat waves and droughts have become more severe and more frequent under elevating CO_2_ concentrations [1]. Plant responses to environmental stress involve multiple adaptations, including those related to morphology (e.g., inhibition of shoot elongation, changes in growth, etc.), physiology (e.g., changes in stomatal conductance, osmotic regulation, etc.), biochemistry (e.g., accumulation of organic and inorganic osmolytes, activation of antioxidant systems and the induction or suppression of metabolic pathways), all of which are based on molecular adaptations [2,3,4]. Abiotic stresses such as drought (D) and elevated temperature (eT) limit many plant functions. One of the most sensitive processes that is crucial for both plant growth and development is photosynthesis [5]. Both steps of photosynthesis (i.e., the electron transport system and the Calvin cycle) are under the control of many genes/gene products that are encoded by the chloroplast and nuclear genome, which work together within a well-coordinated photosynthetic network of genes and regulatory components. Gene expression is highly variable and is influenced by various environmental factors [6]. As a rule, environmental stresses (e.g., drought, unfavorable temperatures, etc.) suppress photosynthesis via stomatal and non-stomatal (metabolic) limitations, the proportions of which may change under different stresses or under combined stresses [7,8].

Many studies have focused on the study of plant responses to individual abiotic factors [3,4,9,10]. Drought is known to result in the loss of water content, reduction in leaf water potential, stomatal conductance and transpiration rate. Wherein, the closure of stomata causes a decrease in CO_2_ diffusion in mesophyll cells and a decrease in the photosynthesis rate [11]. Stomata regulate a balance of photosynthetic CO_2_ uptake and transpiration water loss using a passive-hydraulic strategy in response to differences in water vapor pressure deficit and a chemo-hydraulic strategy associated with abscisic acid (ABA) synthesis [4,10,12,13]. Physiological studies of plant heat effects have identified a significant role of metabolic limitation in reducing apparent photosynthesis intensity, including a decrease in the Rubisco content and its expression, as well as a decrease in the maximum quantum yield of PSII, which is associated with an increased sensitivity of thylakoid membranes to eT [9]. When PSII is damaged and linear electron transport (LET) is blocked, this causes a decrease in the energy supply to chloroplasts. Consequently, this causes activation of the cyclic electron transport (CET) around PSI, which in turn stimulates ATP synthesis. This, coupled with cyclic photophosphorylation, is required for the Calvin cycle and thereby helps plants to withstand stress [14,15]. High temperatures also promote stomatal opening for increased thermoregulation [4,16] and cause glycolate pathway (photorespiration) activation and ROS formation [7,17].

Unlike drought and elevated temperatures, elevated levels of CO_2_ (eCO_2_) usually stimulate photosynthesis intensity—especially in C_3_ plants—by increasing the availability of the CO_2_ substrate in the Calvin cycle [18,19], as well as by enhancing the carboxylase and decreasing the oxygenase functions of Rubisco [20,21]. Moreover, some studies have found that an increase in apparent photosynthesis, as well as a decrease in stomatal conductance and transpiration rate, can lead to a significant increase in leaf water use efficiency (WUE) at eCO_2_ [22]. However, long-term experiments with eCO_2_ have cast doubt on whether eCO_2_ is also associated with the stimulation of photosynthesis [23,24]. It has been suggested that decreased photosynthesis under eCO_2_ may be associated with sugar accumulation, which leads to a decrease in the Rubisco content and activity [25,26] and/or to imbalances in the leaf source–sink system [27,28]. In addition, low photosynthetic rates may result from a long-term decline in stomatal conductance [29,30,31] or from the suppression of photorespiration under lower nitrogen availability, both of which result in reduced photochemical efficiency and reduced photorespiratory defense [20]. Thus, the question of how eCO_2_ affects plants with C_3_ photosynthesis remains open.

Climate change often manifests itself via the combined effects of eCO_2_, high environmental temperature and drought. The synergistic effect of these climatic factors suggests that their combined effect on plant physiology may be much greater than the effect of any individual stressor and, therefore, that studying individual effects may not provide insight into the effects exerted by combinations of stresses, such as those that are experienced under climate change [4,16,32,33]. Plant resistance to combinations of environmental factors is based on complex multigene mechanisms that can have common and sometimes opposing characteristics and cause different effects on plant growth and development [32,34,35]. For example, drought and elevated temperatures can cause similar types of damage to plant cells via oxidative stress and dehydration [4,36,37]. However, the combination of these factors can also cause contradictory behavior with respect to stomatal regulation [38,39,40]. In particular, drought is known to cause a decrease in water potential and a simultaneous increase in the ABA content, which causes stomatal closure. In contrast, heat stress also stimulates stomatal opening to enhance transpirational leaf cooling [4,16,41]. Moreover, despite the contradictory effects of individual factors under combinations of osmotic and temperature stress, stomata generally remain closed; that is, stomatal cells are most strongly controlled by drought, which takes precedence over heat stress responses [4,39,42]. eCO_2_ further complicates the regulation of water metabolism since although eCO_2_ causes most plants to close their stomata, thereby limiting conductivity and water loss [43], according to some authors, stomata can also remain open to improve thermoregulation [38,44]. In combination with these factors, eCO_2_ further complicates the regulation of water metabolism since, under eCO_2_, most plants close their stomata, thereby limiting conduction and water loss [43]. In contrast, some authors suggest that stomata may remain open to improve thermoregulation [38,44]. In addition, eCO_2_ can also mitigate the effects of drought by increasing WUE and improving PSII function [4,45]. Moreover, in combination with temperature stress, eCO_2_ can also accelerate plant growth [46]. However, both high and ultra-high concentrations of CO_2_ are known to exert a negative effect on photosynthesis via drought and heat stress [20,47]. Thus, combined stresses often modify the actions of individual factors and can, therefore, lead to novel effects and molecular reactions based on characteristic transcriptomic responses [41]. These adaptive reactions require further study involving a more detailed understanding of the characteristics of particular plant species, including their tolerance or susceptibility to different combinations of factors [4].

Quinoa (*Chenopodium quinoa* Willd.) is a plant of South American origin that has recently gained widespread recognition for its nutritional properties for humans. Quinoa is grown throughout the world and is characterized by its adaptability to adverse climatic conditions [48]. In this study, we investigate the physiological, biochemical and molecular genetic responses of *C. quinoa* to the individual and combined effects of drought and elevated temperature under ambient (400 ppm, aCO_2_) and elevated (800 ppm, eCO_2_) CO_2_ concentrations. Therefore, we examined the effects of drought, eT and eCO_2_ on photosynthesis and respiration, including photosystem efficiency, CO_2_/H_2_O gas exchange, photosynthetic and photorespiratory enzyme content and gene expression and antioxidant system activity. Specifically, we aimed to (1) confirm or refute hypotheses regarding the synergistic effect of combined action of eCO_2_, eT and D on *C. quinoa* plants and the priority of plant responses to drought regarding eT and eCO_2_; (2) identify protective mechanisms that play a key role in responses to the individual and combined action of factors; (3) determine which parameters can be used as markers of individual and combined stress in *C. quinoa*.

## 2. Results

### 2.1. Growth and Water-Ion’s Balance Parameters

**Figure 1:**
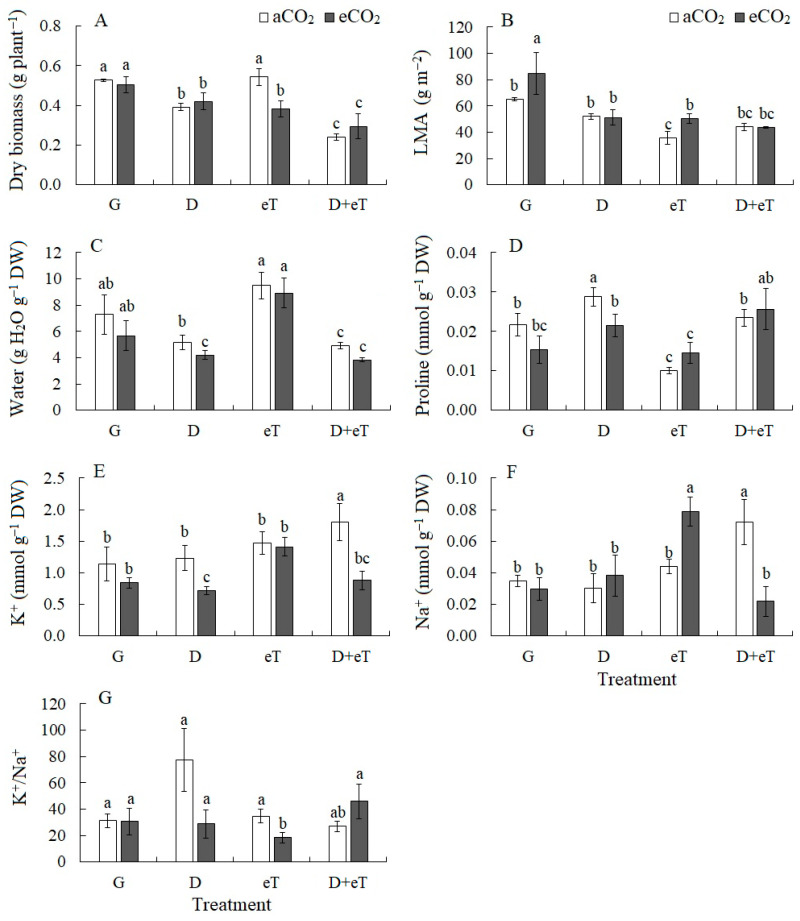
The effect of drought, elevated temperature and combined factors on growth and water-ionic parameters in *Chenopodium quinoa* plants under ambient (400 ppm, aCO_2_) and elevated (800 ppm, eCO_2_) CO_2_ concentrations. (**A**) Dry biomass; (**B**) leaf mass per area, LMA; (**C**) water content; (**D**) proline content; (**E**–**G**) K^+^ and Na^+^ content. G—plants growing at aCO_2_ (control) or eCO_2_ without treatment; D—drought treatment; eT—elevated temperature treatment; D+eT—combined treatment with drought and elevated temperature. Values are means ± standard errors (*n* = 5). The different letters show statistically different means at *p* ≤ 0.05 (Tukey test).

Drought decreased the dry biomass accumulation of *C. quinoa* plants by 25% (Figure 1A). Elevated CO_2_ concentrations (eCO_2_) did not affect growth parameters and did not change drought effects on plants. Elevated temperature (eT) did not have a significant effect on plant growth, but the combined action of eCO_2_ and eT decreased dry biomass accumulation by 28% relative to the control (aCO_2_). The combined action of D+eT and eCO_2_+D+eT decreased dry biomass accumulation by almost 2-fold (Figure 1A). Drought did not cause changes in leaf mass per area (LMA), whereas eCO_2_ increased LMA by 30%, and eT decreased it by 1.8-fold (Figure 1B). Drought caused a slight decrease in the leaf water content, but the combined action of factors (eCO_2_+D, D+eT and eCO_2_+D+eT) decreased it by 1.7-, 1.5- and 1.9-fold, respectively (Figure 1C). Drought increased proline accumulation (by 32%), whereas eT and eCO_2_+eT decreased proline content by 54% and by 32%, respectively (Figure 1D). The K^+^ content decreased under eCO_2_+D (by 39%) and increased under D+eT (by 58%) (Figure 1E). The Na^+^ accumulation increased 2-fold under the combined action of eCO_2_+eT and D+eT (Figure 1F). The K^+^/Na^+^ ratio increased significantly under drought (by 2.5-fold) and decreased under eCO_2_+eT (by 1.7-fold), due to a significant increase in the Na^+^ content (Figure 1G).

### 2.2. Cyclic Electron Transport around PS I (PSI CET) and Efficiency of PS II

**Figure 2:**
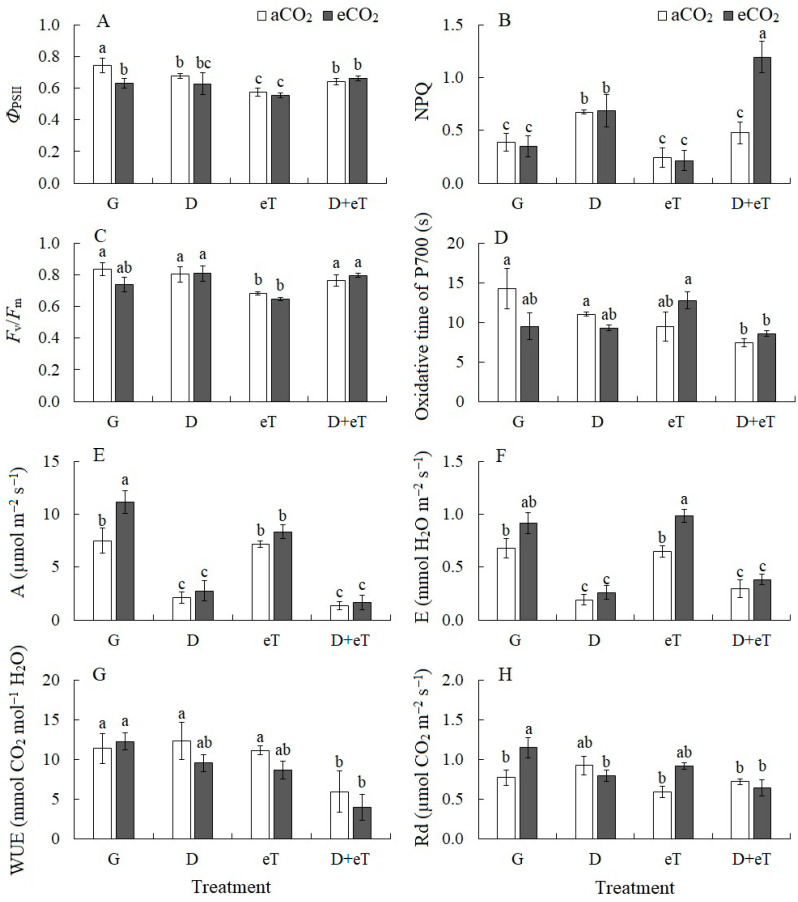
The effect of drought, elevated temperature and combined factors on photosynthetic and respiration parameters in *Chenopodium quinoa* plants under ambient (400 ppm, aCO_2_) and elevated (800 ppm, eCO_2_) CO_2_ concentrations. (**A**) Effective quantum yield of PSII at given light intensities, *Φ*_PSII_; (**B**) non-photochemical quenching of chlorophyll *a* fluorescence, NPQ; (**C**) maximum quantum yield of PSII, *F_v_*/*F_m_*; (**D**) time required to reach the maximum P700 oxidation level under far-red light (PSI); (**E**) apparent photosynthesis, A; (**F**) transpiration intensity, E; (**G**) water use efficiency, WUE; (**H**) dark respiration, Rd. G—plants growing at aCO_2_ (control) or eCO_2_ without treatment; D—drought treatment; eT—elevated temperature treatment; D+eT—combined treatment with drought and elevated temperature. Values are means ± standard errors (*n* = 5). The different letters show statistically different means at *p* ≤ 0.05 (Tukey test).

Drought caused a slight decrease in the effective quantum yield of PSII fluorescence (*Φ*_PSII_) in *C. quinoa* plants at aCO_2_ (by 10%) and in combination with elevated CO_2_ and temperature (eCO_2_+D and eCO_2_+D+eT) by 16% and 12%, respectively (Figure 2A). This was associated with an increase in non-photochemical fluorescence quenching (NPQ) or dissipation costs (by 72%, 77% and 3-folds, respectively) (Figure 2B). Elevated temperature both at aCO_2_ and eCO_2_ decreased the maximum quantum yield of PSII (*F_v_*/*F_m_*) in *C. quinoa* (by 18% and 22%, respectively) (Figure 2C). These changes were accompanied by a decrease in *Φ*_PSII_ (by 23% and 25%, respectively) (Figure 2A). However, under elevated temperatures (eT, eCO_2_+eT, D+eT), as well as at eCO_2_, a decrease in *Φ*_PSII_ (by 23%, 25%, 15% and 16%, respectively) was not accompanied by changes in NPQ (Figure 2A,B).

The combined action of factors (eT+D and eCO_2_+D+eT) decreased the activity of cyclic electron transport around PSI (PSI CET) (by 1.9- and 1.7-fold, respectively) in *C. quinoa* plants (Figure 2).

### 2.3. Intensity of CO_2_/H_2_O Gas Exchange

Drought alone and in combination with other factors (eCO_2_+D, D+eT, eCO_2_+D+eT) reduced the intensity of apparent photosynthesis (A) in *C. quinoa* by 3.6-, 2.8-, 5.8- and 4.7-fold, respectively. The decrease in A under drought was associated with stomatal closure (stomatal limitation) because transpiration intensity (E) showed a similar decrease by 3.5-fold (Figure 2F). With the combined effects of eCO_2_+D, D+eT, eCO_2_+D+eT, the decrease in E was less pronounced (2.3, 2.3 and 1.7 times, respectively). Growing plants at eCO_2_ increased A by almost 50%. Whereas eT did not affect it (Figure 2E). Whereas eCO_2_ and eT led to an increase in E (by 45%) and, consequently, stomata opening (Figure 2F). Water use efficiency (WUE), calculated based on CO_2_/H_2_O gas exchange data (A/E), significantly decreased under the combined stresses (D+eT) and (eCO_2_+D+eT) by 1.9- and 2.9-fold, respectively (Figure 2G). The intensity of dark respiration (Rd) increased (by 37%) under eCO_2_ only (Figure 2H)

### 2.4. Expression of Genes for Components of Light and Dark Reactions of Photosynthesis

**Figure 3:**
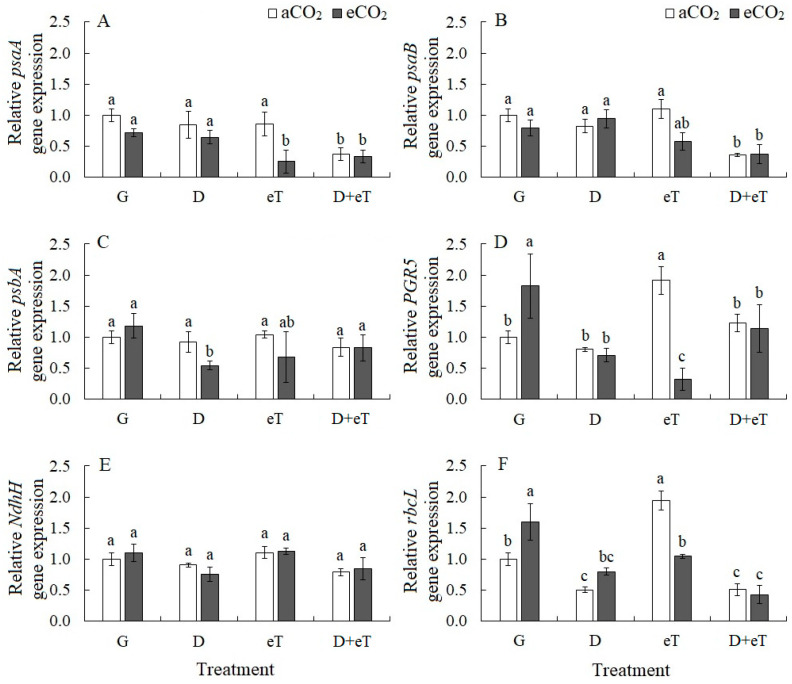
The effect of drought, elevated temperature and combined factors on photosynthetic gene expression in *Chenopodium quinoa* plants under ambient (400 ppm, aCO_2_) and elevated (800 ppm, eCO_2_) CO_2_ concentrations. (**A**,**B**) *psaA* and *psaB*, genes encoding apoproteins 1 and 2 of PSI; (**C**) *psbA*, gene encoding protein D1 of PSII; (**D**) *PGR5*, gene encoding PGR5 protein of the main cyclic electron transport (CET) pathway of PSI; (**E**) *NdhH*, gene encoding the H subunit of the NADH dehydrogenase in the second CET pathway of PSI; (**F**) *rbcL*, gene encoding the Rubisco large subunit. G—plants growing at aCO_2_ (control) or eCO_2_ without treatment; D—drought treatment; eT—elevated temperature treatment; D+eT—combined treatment with drought and elevated temperature. Values are means ± standard errors (*n* = 5). The different letters show statistically different means at *p* ≤ 0.05 (Tukey test).

The expression of genes encoding components of PSI and PSII, *psaA*, *psaB* and *psbA*, as well as the *PGR5* and *NndhH* genes associated with the two pathways of cyclic electron transport (PGR5/PGRL1 and NDH), was studied (Figure 3A–E). The combined stresses D+eT and eCO_2_+D+eT downregulated the expression of *psaA* and *psaB* genes encoding apoproteins A1 and A2 of PSI (by 2.5-fold, Figure 3A,B). eCO_2_+eT downregulated *psaA* expression (3.3-fold). Drought at eCO_2_ (eCO_2_+D) led to a 2-fold decrease in the expression of the *psbA* gene encoding the PSII protein D1 (Figure 3C). The individual effect of eCO_2_ or eT upregulated the expression of the PGR5 gene (by 1.8- and 1.9-fold, respectively), encoding the key protein PGR5 of the main PSI CET pathway (Figure 3D). Whereas the combined action of eCO_2_+eT downregulated *PGR5* expression (by 3-fold). Wherein the expression of the *NdhH* gene, encoding the H subunit of NADH dehydrogenase of the second CET pathway of PSI, did not change under any treatments (Figure 3E). Drought alone and in combination with other factors (D+eT, eCO_2_+D+eT) downregulated the expression of the *rbcL* gene encoding the large subunit of Rubisco (by 2-fold) in *C. quinoa* plants (Figure 3F). Whereas eCO_2_ and eT upregulated *rbcL* expression by 1.6- and 1.9-fold, respectively.

### 2.5. Content of Photosynthetic Enzymes

**Figure 4:**
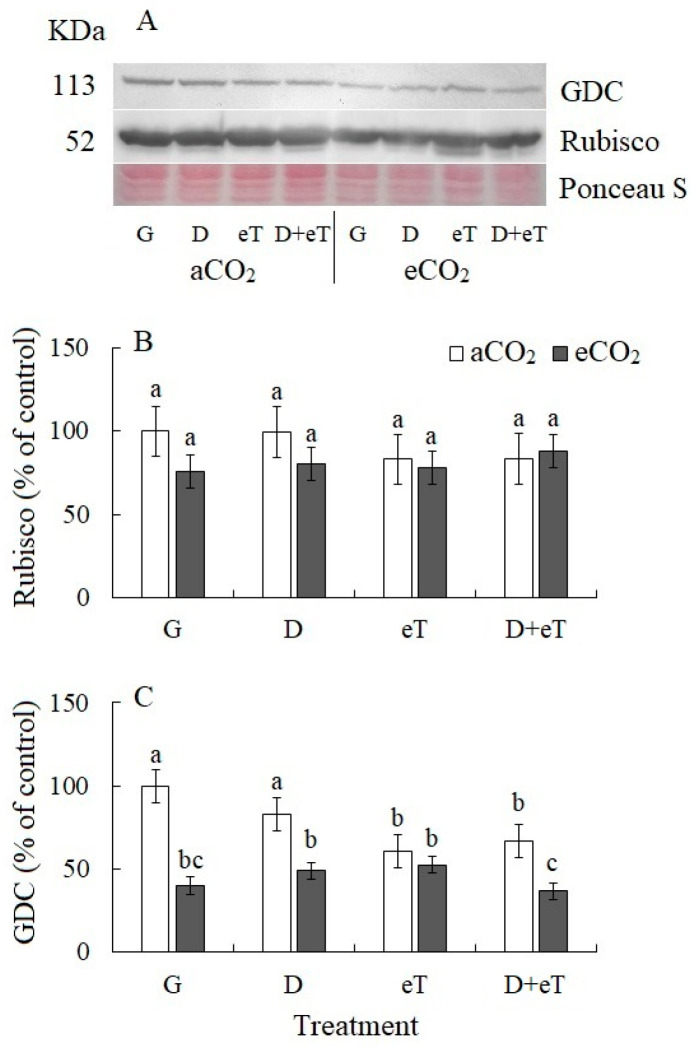
The effect of drought, elevated temperature and combined factors on photosynthesis enzyme content in *Chenopodium quinoa* plants under ambient (400 ppm, aCO_2_) and elevated (800 ppm, eCO_2_) CO_2_ concentrations. (**A**) Western blots for photosynthetic enzymes from soluble total proteins extracted from leaves of *C. quinoa* plants, (**B**) Ribulose-1,5-bisphophate carboxylase/oxygenase (Rubisco, subunit L), (**C**) Glycine decarboxylase (GDC P protein). Equal protein loading was checked by staining the blots with Ponceau. G—plants growing at aCO_2_ (control) or eCO_2_ without treatment; D—drought treatment; eT—elevated temperature treatment; D+eT—combined treatment with drought and elevated temperature. Values are means ± standard errors (*n* = 5). The different letters show statistically different means at *p* ≤ 0.05 (Tukey test).

The content of the main photosynthetic enzyme Rubisco did not change among all treatments of *C. quinoa* (Figure 4A). eCO_2_ alone and in combination with other factors (eCO_2_+eT, eCO_2_+D, eCO_2_+D+eT) decreased the content of the glycolate pathway enzyme (photorespiration) glycine decarboxylase GDC by 60% 51%, 47% and 63%, respectively (Figure 4B). The GDC content was sensitive to temperature effects and decreased at eT and D+eT by 39% and 33%, respectively (Figure 4).

### 2.6. Pro/Antioxidant Balance Parameters

**Figure 5:**
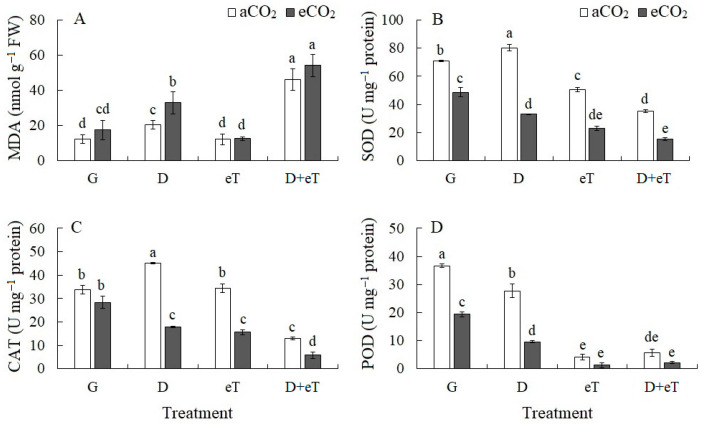
The effect of drought, elevated temperature and combined factors on the lipid peroxidation and antioxidant enzyme activity in *Chenopodium quinoa* plants under ambient (400 ppm, aCO_2_) and elevated (800 ppm, eCO_2_) CO_2_ concentrations. (**A**) malondialdehyde content, MDA; (**B**) superoxide dismutase activity, SOD; (**C**) catalase activity, CAT; (**D**) guaiacol peroxidase activity, POD. G—plants growing at aCO_2_ (control) or eCO_2_ without treatment; D—drought treatment; eT—elevated temperature treatment; D+eT—combined treatment with drought and elevated temperature. Values are means ± standard errors (*n* = 5). The different letters show statistically different means at *p* ≤ 0.05 (Tukey test).

Drought caused a 1.7-fold increase in the content of malondialdehyde (MDA), an indicator of oxidative stress, in *C. quinoa* plants (Figure 5A). Combined stresses eCO_2_+D, D+eT and eCO_2_+D+eT led to even greater increases in MDA content (2.7-, 3.8- and 4.5-fold, respectively). One of the important enzymes of antioxidant defense is superoxide dismutase (SOD), which acts as the first line of defense against ROS, neutralizing the superoxide radical with the formation of hydrogen peroxide. A slight increase in SOD activity (by 13%) was observed under drought (Figure 5B). Other treatments decreased the SOD activity (Figure 5B). Individual actions of eCO_2_ and eT decreased (by 30%) the SOD activity. Combined stresses eCO_2_+D, eCO_2_+eT and D+eT more significantly decreased the SOD activity (by 2–3-fold), whereas eCO_2_+D+eT caused a 4.6-fold decrease in SOD activity. The second line of defense against ROS, namely hydrogen peroxide scavenging, includes catalase (CAT) and guaiacol peroxidase (POD). Drought increased (by 33%) CAT activity, whereas the combined stresses eCO_2_+D, eCO_2_+eT, D+eT and eCO_2_+D+eT decreased it by 1.9-, 2.2-, 2.6- and 5.7-fold, respectively (Figure 5C). POD activity decreased under all treatments (Figure 5D). Individual action of drought and eCO_2_ decreased POD activity by 23% and 1.9-fold, respectively, whereas eT decreased it up to 9 times. The combined stresses eCO_2_+D and D+eT decreased POD activity by 3.8- and 6.4-fold, respectively, and eCO_2_+eT and eCO_2_+D+eT more decreased significantly by 28- and 16.6-fold, respectively,

### 2.7. Multivariate Principal Component Analysis (PCA)

**Figure 6:**
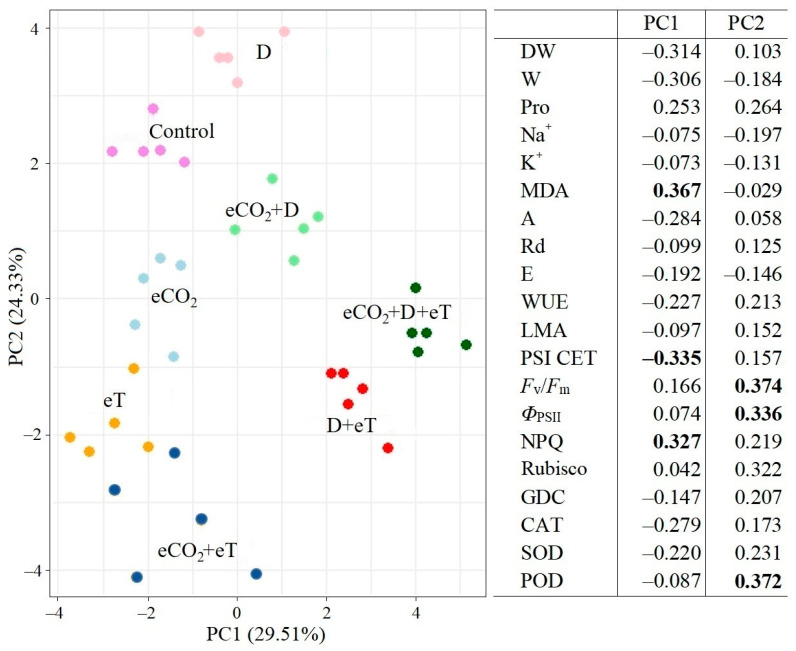
Principal component analysis (PCA) of the physiological data of *Chenopodium quinoa* plants under drought, elevated temperature and combined factors and ambient (400 ppm, aCO_2_) and elevated (800 ppm, eCO_2_) CO_2_ concentrations during cultivation. Control—plants growing at aCO_2_ without treatment; D—drought treatment at aCO_2_; eT—elevated temperature treatment at aCO_2_; D+eT—combined treatment with drought and elevated temperature at aCO_2_; eCO_2_—plants growing at eCO_2_ without treatment; eCO_2_+D—drought treatment at eCO_2_; eCO_2_+eT—elevated temperature treatment at eCO_2_; eCO_2_+D+eT—combined treatment with drought and elevated temperature at eCO_2_. The most significant parameter values for the first principal component (PC1) and the second principal component (PC2) are highlighted in bold.

To assess the degree of changes in the characteristics of the photosynthetic apparatus and adaptive mechanisms under drought, elevated temperature and different CO_2_ concentrations, a PCA of biochemical and physiological parameters of *C. quinoa* was carried out (Figure 6). PCA showed that plant response to drought, regardless of CO_2_ concentration, had a similar trend by both the first principal component (PC1) and second principal component (PC2). Significant factors for PC1 were MDA content, PSI CET activity and NPQ, whereas significant factors for PC2 were PSII efficiency and POD activity. *C. quinoa* plants at eCO_2_ and eT differed from the control ones by PC2. The most significant changes were observed under combined stresses (D+eT, eCO_2_+D+eT); wherein an increase in drought effect was observed (PC1). The first two PC components are sufficient to explain 53.84% of the changes in the total variation.

## 3. Discussion

**Figure 7:**
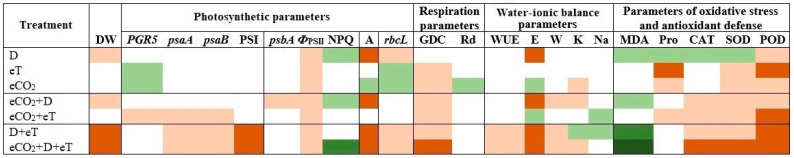
Changes in physiological, biochemical and molecular genetic parameters under individual and combined effects of drought, elevated temperatures and ambient (400 ppm, aCO_2_) and elevated (800 ppm, eCO_2_) CO_2_ concentrations. D—drought treatment at aCO_2_; eT—elevated temperature treatment at aCO_2_; D+eT—combined treatment with drought and elevated temperature at aCO_2_; eCO_2_—plants growing at eCO_2_ without treatment; eCO_2_+D—drought treatment at eCO_2_; eCO_2_+eT—elevated temperature treatment at eCO_2_; eCO_2_+D+eT—combined treatment with drought and elevated temperature at eCO_2_. Green means an increase in the parameters, red means a decrease in the parameters. Dark green and dark red mean a strong increase and decrease in parameters, respectively.

During adaptation to climatic factors, plants undergo changes in their physiological and metabolic characteristics that help to maintain the highest possible level of productivity at the smallest cost [4,10]. Moreover, most plants exhibit both universal and species-specific responses [4,49]. Our studies of *C. quinoa* plants showed that the most severe stress symptoms were induced by drought, both by itself and in combination with elevated temperature (D+eT), and with elevated CO_2_ and eT together (eCO_2_+D+eT) (Figure 7).

### 3.1. Individual Effect of Drought

Under drought, *C. quinoa* plants exhibited decreased dry weight, lower efficiency of light and dark reactions of photosynthesis (i.e., *Φ*_PSII_, apparent photosynthesis, expression of the *rbcL* gene, which encodes the large subunit of Rubisco), as well as transpiration intensity (Figure 1, Figure 2 and Figure 3). Furthermore, decreases in PSII efficiency were accompanied by increased non-photochemical quenching (NPQ), a process that controls the distribution of excitation energy between the reaction center and the antenna complex. By dissipating excess energy as heat, this process protects the photosynthetic apparatus from overexcitation and subsequent damage [5,50]. Moreover, increased NPQ values, higher MDA and proline contents and higher levels of antioxidant enzyme (e.g., CAT and SOD) activity indicate the instability of *C. quinoa* plants with respect to drought, as evidenced by the development of oxidative stress and by activation of the antioxidant defense system (Figure 1, Figure 2 and Figure 5). Similar results indicating the sensitivity of C_3_ species to osmotic stress have been previously demonstrated by other researchers [7,51]. Finally, a strong decrease in A and E intensity in *C. quinoa* indicates that stomatal regulation plays a significant role in photosynthesis limitation under drought. In C_3_ species, stomatal closure is known to be an early response to drought, i.e., decreased stomatal conductance helps maintain plant water balance [4,10]. We speculate that this may be why the water content of *C. quinoa* plants did not change under drought conditions (Figure 1).

### 3.2. Individual Effect of Elevated Temperature

Elevated temperature caused decreased PSII efficiency (*Φ*_PSII_) in *C. quinoa*, which was accompanied by the upregulation of *PGR5*, a gene involved in the PSI CET (Figure 2 and Figure 4). The PSI CET comprises two distinct pathways: one that is dependent on PGR5/PGRL1; and a second that is dependent on the chloroplast NADH dehydrogenase complex [52,53]. In C_3_ species, the PGR5/PGRL1 pathway is considered to be the main pathway and is known to play an important role in plant responses to abiotic stress [53,54]. In addition, eT treatments were observed to affect the balance of the synthesis and degradation of the key photosynthetic enzyme Rubisco, as evidenced by the upregulation of *rbcL* expression but the stability of the Rubisco content under eT (Figure 4). It has been previously shown in other plant species that high-temperature stresses can cause a decrease in Rubisco content and in the expression of its genes [7]. In contrast, moderately high temperatures may improve the photosynthetic parameters associated with achieving maximum productivity under unfavorable conditions [55]. Thus, under eT, *C. quinoa* maintained stable Rubisco content, and damage to PSII and an associated decrease in LET efficiency may be compensated by a shift towards the activation of the main pathway of PSI CET (Figure 7). This ensures that there is an increase in the energy supply to chloroplasts under high-temperature stress [15] and protects against the effects of excess ROS production and the damage caused by oxidative stress [14].

### 3.3. Individual Effect of Elevated CO_2_ Concentration

Under elevated CO_2_, there was an increase in A intensity and a decrease in photorespiration, as measured by the content of glycine decarboxylase (GDC), an enzyme involved in the glycolate pathway that is associated with increased Rubisco carboxylase activity and decreased oxygenase activity. This was accompanied by an increased expression of *rbcL* (Figure 4). eCO_2_ also stimulated the intensity of dark mitochondrial respiration in *C. quinoa*, and this effect was probably associated with additional adaptation or dissipation costs since this treatment was not associated with an increase in growth parameters (Figure 7). In addition, eCO_2_ induced morphological changes in *C. quinoa*, including an increase in leaf mass per area (LMA); this finding is consistent with those of other authors [56]. This parameter indirectly characterizes the thickness and density of the leaf blade, as well as an increase in the growth of non-photosynthetic leaf tissues [57]. Moreover, LMA is genetically determined but can depend on external factors and is therefore thought to be an important indicator of species fitness [57]. Overall, eCO_2_, like eT, reduced PSII efficiency and upregulated *PGR5* expression (Figure 2 and Figure 3). Thus, in *C. quinoa* plants under eCO_2_ or eT, a protective-compensatory mechanism plays an important role; specifically, a decrease in PSII efficiency was accompanied by a shift toward the activation of PGR5/PGRL1-dependent PSI CET, which is involved in various adaptation processes [53,54].

### 3.4. Combined Effect of eCO_2_ with Drought or Elevated Temperature

Next, we examined the response of *C. quinoa* to the combined effect of eCO_2_ and drought (eCO_2_+D). This was in many ways similar to the response to the individual effect of drought, e.g., the closure of stomata led to a decrease in A intensity and PSII efficiency (*Φ*_PSII_) as well as increased NPQ and MDA content values. These effects indicated damage to PSII and were associated with an increase in oxidative stress and a decrease in dry biomass (Figure 7). Another feature of the combined effect of eCO_2_+D was a decrease in water content and the significant downregulation of *psbA* expression, a gene encoding the PSII D1 protein, which plays an important role in restoring PSII damage incurred by abiotic stress [58].

A unique response caused by a combined effect of eCO_2_ and elevated temperature (eCO_2_+eT) was the downregulation of *PGR5* expression. This is significant since the individual action of both factors was the upregulation of its expression (Figure 7). Thus, under the combined eCO_2_+eT stress, we observed a decrease in PSII efficiency that was not accompanied by a shift toward the activation of the PGR5/PGRL1-dependent CET of PSI, which is known to play a protective and adaptive role [14,52,53]. Moreover, significant damage to PSI under CO_2_+eT was also evidenced by the suppression of the expression of *psaA* and *psaB*, two genes of PSI (apoproteins A1 and A2) (Figure 7). Therefore, individual and combined factors act differently on *C. quinoa* plants and can cause unexpected effects. These are likely associated with a transcriptomic response unique to each combination of factors [40,42]. PCA showed that eT affects *C. quinoa* plants more strongly at aCO_2_ than at eCO_2_; that is, plants grown under eCO_2_ conditions showed weaker responses to eT (Figure 6). This finding is consistent with data from other studies regarding the mitigating effect of eCO_2_ on the negative effects of thermal stress [45].

### 3.5. The Combined Effect of Drought with Elevated Temperature at aCO_2_ and eCO_2_

The combined effect of drought and elevated temperature usually has a more negative effect on a plant than either effect alone [4,59]. This trend was confirmed to be true in *C. quinoa* during our experiments (Figure 7). For example, decreases in many parameters of *C. quinoa* under D+eT (e.g., dry biomass, intensity of light and dark reactions of photosynthesis, transpiration and *rbcL* expression) are similar to the response to drought, i.e., water deficit played a critical role in plant responses, another finding that is consistent with those of other authors [4,39,42].

In general, strong decreases in apparent photosynthesis, compared with transpiration, may indicate the presence of both stomatal and metabolic limitations of photosynthesis. This can lead to a noticeable decrease in WUE (Figure 1). A characteristic feature of the plant response to D+eT was a decrease not only in the functioning of PSII (*Φ*_PSII_) but also PSI, which is generally considered to be more stress-resistant [60]. Moreover, this effect may be due to the decreased expression of *psaA* and *psaB*. Taken together, these data indicate that there was significant disruption of the function of the photosynthetic ETC. However, we also observed no activation of any PSI CET pathways, which are known to play an important role in antioxidant protection and ensuring photosynthetic activity under stress [14] (Figure 2 and Figure 4). Moreover, photorespiration can play a significant protective role during stress since it protects the photosynthetic apparatus from photoinhibition by using excess NADPH and ATP that are formed during the light phase of photosynthesis, thereby preventing the over-reduction of chloroplasts [61]. However, in our experiments, we observed that the combined action of D+eT, as well as the individual action of eT decreased GDC content in *C. quinoa* (Figure 4) and may indirectly also indicate a decrease in the photorespiration intensity. Overall, our data showed that the combined effect of D+eT caused a change in the adaptive strategy and a decrease in the activity of some protective mechanisms relative to individual actions, which led to significant oxidative stress and a fourfold increase in MDA content.

The combined effect of three factors (i.e., eCO_2_+D+eT) also led to significant tissue dehydration in *C. quinoa,* as at D+eT (Figure 1). A significant increase in NPQ, a decrease in PSII efficiency, PSI CET activity, *psaA* and *psaB* gene expression, and the lack of activation of *PGR5* expression (i.e., a PSI CET gene) together indicated a significant disruption of ETC functioning, which is known to be accompanied by intense ROS formation [62]. Here, an increase in oxidative stress (i.e., up to 4.5-fold increased MDA) in *C. quinoa* was accompanied by a decrease in the activity of antioxidant enzymes (CAT, SOD, POD) and in the photorespiratory protein (GDC) content (Figure 2 and Figure 4). Multivariate analysis (PCA) confirmed that the response of *C. quinoa* to the combined stresses D+eT and eCO_2_+D+eT was the strongest (Figure 6). The most significant processes in plants affected by the combined action of two or three factors included an increase in MDA content and NPQ values, as well as a decrease in PSI CET activity and dry biomass (Figure 6). Moreover, an increase in MDA, NPQ, and a decrease in dry biomass were also all observed under drought alone but were significantly intensified in combined treatments.

Taken together, the results obtained for *C. quinoa* confirmed the priority of key plant responses to water deficiency over thermal effects and eCO_2_. Moreover, a decrease in PSI CET activity and WUE was observed only under combined stresses D+eT and eCO_2_+D+eT (Figure 7) and the combined action of these three factors led to significant oxidative stress and dissipative losses associated with the disruption of PSI and II. Moreover, the absence or decrease in the functioning of protective mechanisms associated with PSI CET, photorespiration and antioxidant enzymes may all cause decreases in the dry biomass of *C. quinoa* plants.

## 4. Materials and Methods

### 4.1. Plant Growth Conditions

Seeds of the Vakhdat variety of *Chenopodium quinoa* Willd. were produced on experimental trials within SATREPS project (JST, JPMJSA2001). Seeds were germinated in distilled water and the seedlings were transplanted to perlite in 8 plastic pots (20 seedlings per pot), which were divided into two separate climate chambers and exposed to diurnal lighting (commercial white light fluorescent bulbs): 10 h dark/14 h light (200 µmol m^−2^ s^−1^ PAR), at 25 ± 2 °C and two levels of CO_2_ concentration: at ambient (400 ppm, aCO_2_) CO_2_ (80 plants) and at elevated (800 ppm, eCO_2_) CO_2_ (80 plants). All plants were grown in 50% Hoagland’s solution for 30 days. Subsequently, 20 plants were treated with a 12.5% (m/v) solution of PEG 6000 for 4 days, 20 plants were treated with elevated temperature (32 ± 2 °C) for 4 days and 20 plants were treated with both elevated temperature and PEG for 4 days. As a control, 20 plants at 50% Hoagland’s solution were used. Altogether 8 experimental variations of plants at the stage of vegetative growth were analyzed: (1) plants growing at aCO_2_ and 25 °C, (Control); (2) plants growing at aCO_2_ and 25 °C + 4 days, treated with PEG (D); (3) plants growing at aCO_2_ and 25 °C + 4 days at elevated temperature (eT); (4) plants growing at aCO_2_ and 25 °C + 4 days treated with PEG and elevated temperature (D+eT); (5) plants growing at elevated CO_2_ (eCO_2_) and 25 °C, (eCO_2_); (6) plants growing at eCO_2_ and 25 °C + 4 days, treated with PEG (eCO_2_+D); (7) plants growing at eCO_2_ and 25 °C + 4 days at elevated temperature (eCO_2_+eT); (8) plants growing at eCO_2_ and 25 °C + 4 days treated with PEG and elevated temperature (eCO_2_+D+eT). Whole plants were used to analyze fresh and dry biomass. To measure the remaining parameters, well-developed leaves from the middle stem tier were used.

### 4.2. Determination of Growth and Water-Ion’s Balance Parameters

The plants were dried at 80 °C until a constant weight was reached in order to determine the water content (W) and leaf dry mass per area (LMA) (Table S1).

Leaf Na^+^ and K^+^ contents were measured in aqueous extracts of 100 mg dry sample using flame photometer FPA-2-01 (Zagorsk Optical-Mechanical Plant, Sergiev Posad, Russia).

The level of free proline was assessed by employing an acid ninhydrin reagent (Table S1).

### 4.3. CO_2_/H_2_O Gas Exchange

The intensity of the CO_2_/H_2_O parameters (apparent photosynthesis (A), transpiration rate (E), dark respiration (Rd) and water use efficiency (WUE)) was measured using a gas analyzer Li-Cor (Inc., Lincoln, NE, USA), as previously described [63] (Table S1).

### 4.4. Efficiency of PSII Function and Activity of Cyclic Electron Transport (CET) around PSI

CET PSI activity was measured as the time required for P700 oxidation in response to far-red illumination using an ED-P700DW pulse-modulated system and PAM 101 fluorometer (Heinz-Walz GmbH, Effeltrich, Germany) [64,65] (Table S1).

The quantum yield of PSII photochemistry (*F_v_*/*F_m_*, *Φ*_PSII_, *NPQ*) in dark-adapted (20 min) leaves was determined using a pulse amplitude-modulated fluorometer (PAM-101, Heinz-Walz GmbH, Effeltrich, Germany) [66] (Table S1).

### 4.5. Content of Photosynthetic Enzymes

Analysis of the content of ribulose-1,5-bisphophate carboxylase/oxygenase (Rubisco) and glycine decarboxylase (GDC) proteins was determined by immunoblot analysis with SDS-PAGE and commercial polyclonal antibodies obtained from Agrisera (Vännäs, Sweden) for proteins of the large subunit (L) of Rubisco (RbcL, AS03037) and glycine decarboxylase P protein (GLDP, AS204370), as described previously [67]. The obtained results were expressed relative to the average level for control plants, which was taken as 100%. The analysis was performed at least 3 times.

### 4.6. Quantitative Real-Time (RT)-PCR

Total RNA was extracted using phenol-chloroform extraction with LiCl precipitation, as described previously [67]. Reverse transcription was carried out according to the standard protocols (Evrogen, Moscow, Russia) and cDNA concentration was measured by a NanoDrop 1000 spectrophotometer (ThermoScientific, Waltham, MA, USA). PCR primers were designed using Pick Primers NCBI and SnapGene Viewer (4.2.11) on nucleotide sequences of *C. quinoa* from the NCBI database (Table S2). Transcript levels were assessed by real-time PCR (RT-qPCR) using a Light Cycler96 amplifier (Roche, Basel, Switzerland) with SybrGreen I dye (Evrogen, Moscow, Russia). RT-PCR data were analyzed using Light Cycler96. Software version 1.1. The level of gene expression was calculated relative to the reference gene, and then the experimental variants were expressed as a percentage with regard to the control, which was taken as 100%.

### 4.7. Assay of Antioxidant Enzyme Activity and Lipid Peroxidation

The activity of superoxide dismutase (SOD; EC 1.15.1.1), peroxidase (POD; EC 1.11.1.7) and Catalase (CAT; EC 1.11.1.6) was measured by spectrometry, as described previously [68] (Table S1).

The intensity of lipid peroxidation was determined by measuring malondialdehyde (MDA) by its reaction with thiobarbituric acid, according to the method of Heath and Packer [69].

### 4.8. Statistical Analyses

All of the measurements were performed on 5 biological and 3 analytical replicates. SigmaPlot v.12.0 software was used for the analysis of variance (ANOVA) with a post hoc Tukey’s test for pairwise comparisons. Means and their standard errors are shown in the figures. Statistical significance (Tukey’s test) was achieved at the *p* < 0.05 level for differences in means between treatments and populations. R v.3.6.1 software was used for principal component analysis (PCA) with multiple factors. The plotting package “ggplot2” was used for multiple factor analysis and grouping of experimental plots under different conditions (PCA). “factoextra” was used for multiple correlations between all parameters of experimental plots under different treatments.

## 5. Conclusions

In this paper, we conducted a comprehensive (i.e., morphophysiological, biochemical and molecular genetic) analysis of the tolerance of *C. quinoa* to the individual and combined effects of abiotic factors such as drought, elevated temperatures and elevated CO_2_ concentration. Despite the fact that this species is characterized by a high adaptability to unfavorable climatic conditions, in our experiments, we demonstrated increased sensitivity to drought and the combined stresses D+eT and CO_2_+D+eT.

At the individual action of drought, we observed an increase in the non-photochemical quenching of chlorophyll fluorescence. This quenching effect dissipates excess energy as heat, thereby protecting PSII from overexcitation and subsequent damage. Under the individual action of eT or eCO_2_, a major role was played by a protective compensation mechanism associated with the upregulation of PGR5, a gene of PSI CET, when PSII functioning was disrupted. A constant Rubisco content under these stresses was ensured by an increase in its biosynthesis, which was confirmed by the activation of *rbcL* expression. Under the combined action of factors, the plant reactions partially repeated the reactions to the individual action of these factors, and the effect of water deficiency was the priority. Under combined stresses, some synergistic effects were also evident, meaning that the cumulative impact of factors on plant physiology was significantly more diverse than that of any individual stress. This is probably due to a change in plant adaptive strategies that facilitate the appearance of some and the loss of other adaptive or protective mechanisms, including PSI CET, photorespiration and antioxidant enzymes, which play a significant role in adaptation to the individual action of each factor. As a result, the combined action of two or three factors, compared with their individual action, led to more significant dehydration, oxidative stress and an increase in dissipative losses associated with the impaired functioning of PSI and II, as well as a decrease in growth parameters.

The most significant or marker parameters related to the response of *C. quinoa* plants to the individual effect of drought included an increase in MDA and NPQ. Under combined stresses D+eT and eCO_2_+D+eT, in addition to these parameters, the activity of PSI CET and dry biomass can be used. Under individual action of eT or eCO_2_, the upregulation of PGR5 and *rbcL* expression was as marker parameter.

## Data Availability

Data is contained within the article.

## Supplementary Materials

The following supporting information can be downloaded at: https://www.mdpi.com/article/10.3390/plants13121666/s1, Table S1: Description of methods and technical procedures, Table S2: List of used primers.
