# Supplementary material for: Drought Has a Greater Negative Effect on the Growth of the C3 Chenopodium quinoa Crop Halophyte than Elevated CO2 and/or High Temperature
Source: Plants (Basel). 2024 Jun 16;13(12):1666. doi: 10.3390/plants13121666 (PMC11207731; doi:10.3390/plants13121666)
Supplement: Supplementary file 1 [file plants-13-01666-s001.zip › plants-3041874-supplementary.pdf]

Table S1. Description of methods and technical procedures

Parameters	Procedure description/formula	Reference
Water content (W)	W (g H_2O/g dry weight) = (FW – DW)/DW, where FW and DW represents the fresh and dry biomass respectively.	[1]
Leaf dry mass per area (LMA)	was quantified as the product of leaf density (LD, g DW cm^{-3} leaf) and the leaf volume to area ratio (LVA, cm^3 m^{-2}) and was calculated according to formula: LMA = LD × LVA.	[2]
Free proline	Dry shoot samples (0.2 g) were homogenized in 2 ml of boiling distilled water, heated at 100°C for 10 min in a water bath, and then the homogenates were centrifuged (5 min, 14,000g). Approximately 1 ml of homogenate was reacted with 1 ml acidic ninhydrin (ninhydrin 1% (w/v) in glacial acetic acid 60% (v/v), 6 M orthophosphoric acid 40% (v/v)), and 1 ml glacial acetic acid in a tube for 1 h at 100°C in a water bath, and the reaction terminated in an ice bath. The mixtures were read at 520 nm using a Genesis 10 UV Scanning spectrophotometer (Thermo Scientific, USA). Proline concentrations were determined using a calibration curve and expressed as mmol DW g−1.	[3]
Apparent photosynthesis (A) and leaf transpiration rate (E)	Leaves were placed in a temperature-controlled (25°C) leafchamber and illuminated with a fiber optic light guide from a KL 1500LCD light source (Schott, Germany) with a halogen lamp (150 W, Philips, Germany) and a PAR intensity of 2000 μmol m-2 s. Steady-state CO2/H2O exchange rates in leaves were measured in open-loop mode using a single-channel infrared gas analyzer (LI-820, LICOR, USA).<br>Leaf transpiration (E) was calculated from the difference in gas humidity at the inlet and outlet of the leaf chamber. The humidity of the gas flow at the inlet of the leaf chamber was maintained at a known level using a LI-610 dew point generator (LI-COR, USA). The moisture content at the outlet of the leaf chamber was determined using a humidity sensor (Vaisala INTERCAP, Finland) HMP50. Atmospheric air was drawn into a 60-L polyethylene gasholder and injected into the chamber at a flow rate of 100 mL/min for continuous operation over 8 hours. The gas circuit's mixing unit maintained a CO_2 concentration of 400 ppm in the airflow supplied to the leaf chamber.	[1]
Dark respiration (Rd)	After measuring photosynthetic CO_2/H_2O exchange, the light was turned off to measure the steady-state dark respiration (Rd) of the leaves.	[1]
Water use efficiency (WUE)	was calculated as the A/E ratio.	
CET PSI activity	Changes in the redox potential of chlorophyll P700 were measured by monitoring leaf absorbance at 820 nm using a dual wavelength pulse-modulated system ED-P700DW (Heinz-Walz GmbH, Effeltrich, Germany) combined with a PAM 101 fluorometer (Heinz-Walz GmbH, Effeltrich, Germany). Far-red light illumination (720 nm, 17.2 W/m2) was used to measure the kinetics of P700 oxidation. The flash of a xenon gas discharge lamp (50 ms, 1,500 W/m2; Walz Effeltrich, Germany) in the presence of far-red light was used to determine the level of maximum P700 oxidation. PSI cyclic electron transport activity was measured as P700 oxidation kinetics in response to far-red illumination by mon. Changes in chlorophyll P700 redox potential were measured by monitoring leaf absorbance at 820 nm using a dual wavelength pulse-modulated system ED-P700DW in combination with a PAM 101 fluorometer (Heinz-Walz GmbH, Effeltrich, Germany).	[4,5]
PSII efficiency	The quantum yield of PSII photochemistry in dark-adapted leaves (20 min) was determined by means of a pulse amplitude modulated fluorometer (PAM-101, Heinz-Walz GmbH, Effeltrich, Germany) [66]. The sample was illuminated with weakly modulated red light during the measurements. The output signal of the PAM-101 was processed by an analogue-digital converter (PDA-100, Heinz-Walz GmbH, Effeltrich, Germany). The signal was displayed on a computer. The maximum quantum yield in the dark-adapted state (F_v/F_m), the effective quantum yield of PSII fluorescence (Φ_{PSII}), and the non-photochemical quenching (*NPQ*) values were calculated using the following equations:	[6]

	$F_v/F_m = (F_m - F_0)/F_m$,<br>$\Phi_{PSII} = F'_q/F'_m$,<br>$NPQ = (F_m - F'_m)/F'_m$,<br>where F_0 and F_m are minimal and maximal chlorophyll fluorescence of a dark-adapted leaf; F'_q is a photochemical quenching of fluorescence by an open reaction center of PSII and F'_m is a maximum chlorophyll fluorescence after light adaptation.	
Protein content	Frozen plant leaves (0.5 g) were homogenized in 0.1 M Tris-HCl (pH 7.4) containing 1 mM dithiothreitol (DTT), 0.5 mM phenylmethylsulfonyl fluoride, and 0.5% dimethyl sulfoxide (DMSO). The homogenates were centrifuged at 10,000 *g* for 15 minutes at 4°C. The supernatant was used to determine the activity of antioxidant enzymes. Protein content was determined using the Bradford method with bovine serum albumin ("Sigma-Aldrich", USA) as the standard.	[7]
SOD	Superoxide dismutase (SOD; EC 1.15.1.1) activity was determined using a reaction mixture: 0.1 M Tris-HCl (pH 7.8), 50 μM nitroblue tetrazolium, 10 mM L-methionine, 0.025% Triton X-100, and 3 μM riboflavin. The reaction was initiated by adding the enzyme extract and exposed to light at an intensity of 350 μmol/(m^2 s). Absorbance was recorded at 560 nm (Multiskan™ Sky, "Thermo Fisher Scientific", USA).	[7,8]
POD	Peroxidase (POD; EC 1.11.1.7) activity was determined by recording the increase in optical density at 470 nm during the oxidation of guaiacol. The reaction mixture contained 0.1 M Tris-HCl (pH 7.4), 7 mM guaiacol, 4 mM H_2O_2, and the enzyme extract.	[7,9]
CAT	Catalase (CAT; EC 1.11.1.6) activity was determined by mixing the enzyme extract with 0.1 M Tris-HCl (pH 7.4) and 0.1 M H_2O_2. Catalase activity was measured by the decrease in optical density at 240 nm for 1 minute.	[7,10]
MDA	The intensity of lipid peroxidation was determined according to the method of Heath and Packer. Malondialdehyde (MDA) was measured by reaction with thiobarbituric acid. Optical densities at 532 nm and 600 nm were measured in the samples. The absorption coefficient of 155 mM^{-1} cm^{-1} was used to calculate the MDA content.	[7,11]

REFERENCES

1. Rakhmankulova, Z.F., Shuyskaya, E.V., Prokofieva, M.Y. *et al.* Effect of elevated CO_2 and temperature on plants with different type of photosynthesis: Quinoa (C_3) and Amaranth **(C_4)**. *Russ. J. Plant. Physiol.* **2023**, *70*, 117. doi: 10.1134/S1021443723601349.
2. Poorter, H.; Niinemets, Ü.; Poorter, L.; Wright, I.J.; Villar, R. Causes and consequences of variation in leaf mass per area (LMA): a meta-analysis. *New Phytol.* **2009**, *182*(3), 565–588. doi: 10.1111/j.1469-8137.2009.02830.x.
3. Shuyskaya, E., Rakhmankulova, Z., Prokofieva, M., et al. Intensity and duration of salinity required to form adaptive response in C4 halophyte *Kochia prostrata* (L.) Shrad. *Front. Plant Sci.* **2022**, *13*, 955880. https://doi.org/10.3389/fpls.2022.955880
4. Klughammer, C.; Schreiber, U. Measuring P700 absorbance changes in the near infrared spectral region with a dual wavelength pulse modulation system, in Garab G. (Ed.), Photosynthesis: Mechanisms and Effects. Kluwer Academic Publishers, Dordrecht, **1998**, p. 4357. https://doi.org/10.1007/978-94-011-3953-3_1008
5. Nakamura, N.; Iwano, M.; Havaux, M.; Yokota, A.; Munekage, Y.N. Promotion of cyclic electron transport around photosystem I during the evolution of NADP-malic enzyme-type c photosynthesis in the genus Flaveria. *New Phytol.* **2013**, *199*, 832–842. https://doi.org/10.1111/nph.12296
6. Schreiber, U. Chlorophyll fluorescence and photosynthetic energy conversion: Simple introductory experiments with the TEACHING-PAM chlorophyll fluorometer; Heinz Walz GmbH: Effeltrich, Germany, **1997**.
7. Shuyskaya E., Rakhmankulova Z., Prokofieva M., Lunkova N., Voronin P. Salinity mitigates the negative effect of elevated temperatures on photosynthesis in the C_3-C_4 intermediate species *Sedobassia sedoides. Plants* **2024**, *13*, 800. doi: 10.3390/plants13060800.
8. Beauchamp, C.; Fridovich, I. Superoxide dismutase: improved assays and an assay applicable to acrylamide gels. Anal. Biochem. **1971**, *44*, 276–287. https://doi.org/10.1016/0003-2697(71)90370-8
9. Shevyakova, N.I.; Stetsenko, L.A.; Meshcheryakov, A.B.; Kuznetsov, Vl.V. The activity of the peroxidase system in the course of stress-induced CAM development, *Russ. J. Plant Physiol.* 2002, 49, 598–604.
10. Aebi, H. Catalase in vitro. Methods Enzymol. **1984**, *105*, 121–126. https://doi.org/10.1016/s0076-6879(84)05016-3
11. Heath, R.L.; Packer, L. Photoperoxidation in isolated chloroplasts, *Arch. Biochem. Biophys.* 1968, 125, 180–198. https://doi.org/10.1016/0003-9861(68)90654-1

Table S2. List of used primers

Primer	Gene ID	Function	5'–3' sequence
rbcL	32958948	Large subunit (L) Rubisco	TCACATGTAGCGGCAGTAGC<br>AGCCGTTTATGCGTTGGAGA
psaA	32958941	Apoprotein A1 of photosystem I	GTGAGTAGGGTCGCTTAGCC<br>TACCAGCGACTTGGAGGAGA
psaB	32958940	Apoprotein A2 of photosystem I	GAACCGCGTGCATCTAAAGC<br>GCCTGGCTGGTTAAATGCTG
psbA	32959011	Protein D1 of photosystem II	AGACCCGGAAACAGGTTCAC<br>ACCAGCACTGAAAACCGTCT
PGR5	110692940	PGR5 protein, a key part of the main CET pathway of PSI	TCACAACCACAAGAGGAGCAA<br>TCGCGTCCGGTGAGAATTAC
NdhH	32959000	H subunit of the NADH dehydrogenase in the second CET pathway of PSI	GGCCATTTCACCGATTCGTA<br>GGCCCTATGCTACGAGCTTC
UBQ10	110721034	Ubiquitin 10 (reference gene)	CGAGCAGAAACAAGCCTAATCG<br>GCGATTAATTTCCATGTTGTCCG

PCR primers were designed using Pick Primers NCBI (National Center for Biotechnology Information, Bethesda, MD) on nucleotide sequences of *Chenopodium quinoa* from NCBI data base. The specificity of the primers was ensured by the function "Primer Pair Specificity Checking Parameters" Pick Primers NCBI. Then all primers were checked using SnapGene Viewer (4.2.11). UBQ10 was used as reference gene.
